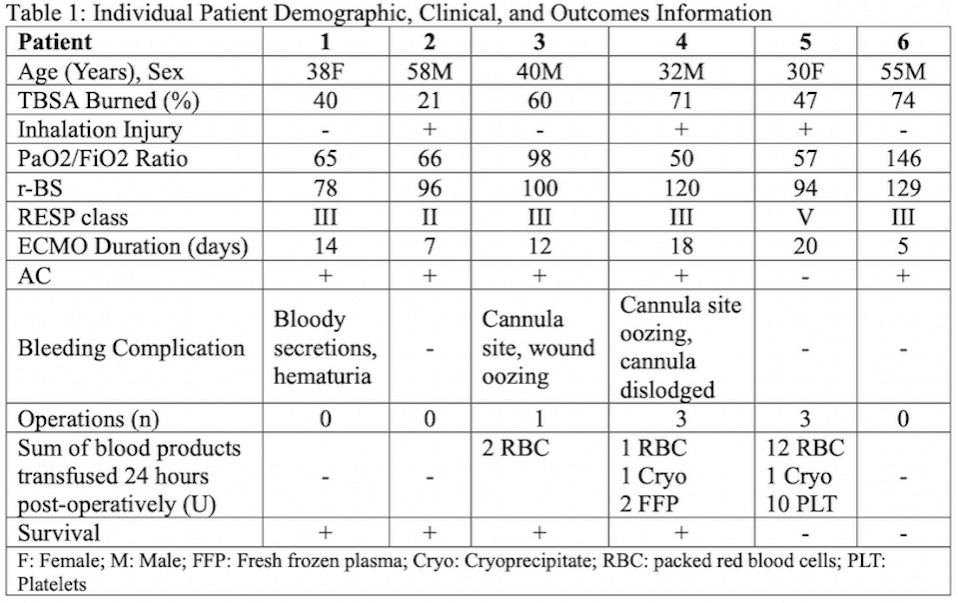# 569 ECMO as Salvage Therapy in Burn-Related ARDS

**DOI:** 10.1093/jbcr/iraf019.198

**Published:** 2025-04-01

**Authors:** Talia Arcieri, Ana Reyes, Jessica Delamater, Michael Cobler-Lichter, Nicholas Namias, Louis Pizano, Joyce Kaufman, Shevonne Satahoo, Kenneth Proctor, Carl Schulman, Brandon Parker

**Affiliations:** Miami Burn Center; University of Miami; University of Miami, Jackson Memorial Hospital; University of Miami, Jackson Memorial Hospital; University of Miami; University of Miami / Jackson Health System; University of Miami; University of Miami; University of Miami Miller School of Medicine; University of Miami; University of Miami

## Abstract

**Introduction:**

Extracorporeal membrane oxygenation (ECMO) is an effective therapy for severe acute respiratory distress syndrome (ARDS), but its use in burn patients is uniquely challenging due to bleeding risk associated with frequent operations. We aimed to characterize a case series of burn patients with the hypothesis that ECMO use is feasible in appropriately selected patients.

**Methods:**

All burn patients treated with ECMO from November 2021 to August 2024 at one burn center were retrospectively reviewed. Revised-Baux scores (r-BS) and Respiratory ECMO Survival Prediction (RESP) classes were calculated.

**Results:**

Six patients (mean burn total body surface area (TBSA) 52.1%, mean r-BS 102.8, RESP class range II to V) received veno-venous ECMO. Three patients had non-surgical bleeding complications (Patients 1, 3, 4; Table 1). Three patients (Patients 3, 4, 5) had operations while on ECMO. Patient 3 underwent bedside burn excision and tracheostomy with anticoagulation (AC) held 3 hours pre-operatively (pre-op), and required 2 units (U) of blood products in the 24-hour post-operative period. Patient 4 underwent two bedside burn excisions with AC held 1 hour pre-op, and OR tracheostomy with AC held 13 hours pre-op, and required a sum of 4U of blood products in the 24 hours following all operations. Patient 5 did not receive AC due to coagulopathy, but had two bedside burn excisions and one OR burn excision with lower extremity amputation, requiring a sum of 23U of blood products in the 24 hours following all operations. Three patients had AC held at any time with no thrombotic complications. Four patients survived (three discharged to acute inpatient rehabilitation, one home). Two patients died of severe sepsis.

**Conclusions:**

ECMO is a feasible salvage therapy in appropriately selected burn patients with ARDS. Bleeding is a concern, but this case series suggests that operative intervention and AC can be safe with close monitoring. Further studies in larger patient populations are needed to identify which phenotypes benefit most from ECMO.

**Applicability of Research to Practice:**

This contributes to the growing scientific understanding of burn patients treated with ECMO and may inform the development of criteria for successful ECMO use in this population.

**Funding for the Study:**

N/A